# Sows-Gilts Stocking Rates and Their Environmental Impact in Rotationally Managed Bermudagrass Paddocks

**DOI:** 10.3390/ani10061046

**Published:** 2020-06-17

**Authors:** Silvana Pietrosemoli, James T. Green, Maria Jesús Villamide

**Affiliations:** 1 Department of Animal Science, College of Agriculture and Life Sciences, North Carolina State University, Raleigh, NC 27695-7621, USA; 2 Departamento de Producción Agraria, E.T.S.I. Agronómica, Alimentaria y de Biosistemas, Universidad Politécnica de Madrid, 28040 Madrid, Spain; mariajesus.villamide@upm.es; 3 Department of Crop and Soil Science, College of Agriculture and Life Sciences, North Carolina State University, Raleigh, NC 27695-7621, USA; jim_green@ncsu.edu

**Keywords:** bermudagrass, sows-gilts, stocking rate, ground cover, soil nutrients, pasture-based pig systems, outdoor pig systems, grazing pigs

## Abstract

**Simple Summary:**

Maintaining a ground cover greater than 75% and controlling nutrient loading and distribution are considered best management practices for pasture pig operations. These practices improve soil health and water quality, by minimizing runoff containing soil, water and nutrients. Those goals are not easily reached when managing pigs on pastures. This study was conducted to evaluate the effect of three sow stocking rates (10, 15 or 25 sows-gilts ha^−1^) on ground cover and soil nutrient concentrations of bermudagrass (*Cynodon dactylon* L. Pers) paddocks managed in a rotational stocking system. Increasing the Stocking rates were inversely related with the deterioration of vegetative ground cover and directly related to soil nutrient loads in the soil. The stocking rates should be kept in the range of 10 to 15 sows-gilts ha^−1^ to minimize the environmental impact of sows-gilts managed on bermudagrass.

**Abstract:**

Ground cover maintenance and nutrients management are key elements to reduce the environmental impact of outdoor swine production. The objective of this study was to determine the effects of sows-gilts stocking rates on vegetative ground cover and soil nutrient concentrations in rotationally stocked bermudagrass (*Cynodon dactylon* L. Pers) pastures. Three stocking rates (10, 15 and 25 sows-gilts ha^−1^) were compared during three 8-week grazing periods. Increasing the stocking rate from 10 to 25 sows-gilts ha^−1^ decreased the ground cover of the paddocks from 65 to 48%, and increased soil nutrient concentrations (ammonium 47%; nitrate 129%; phosphorus 53%; zinc 84%; and copper 29%).

## 1. Introduction

Meat consumers have shown an increasing interest in purchasing products from more sustainable production systems that are considered more respectful of the environment and of animal welfare [1,2]. Those systems reduce the use of agrochemicals and fossil fuels, encourage the rescue of local animal genetic resources, and contribute to the strengthening of local communities [3]. This reorientation promotes the creation and consolidation of niche markets. Niche pork production systems have the capability of including the attributes that more informed consumers are demanding, and to become a more sustainable approach to meat production [4]. Pasture pig systems fit well into productive systems to provision niche markets, as observed with the renowned popularity achieved by “Iberico” products from Spain. This traditional system is found in a unique ecosystem of the Iberian Peninsula called “La Dehesa”. In the Dehesa, pigs of the ‘Iberico’ heritage breed are managed in a silvopastoral system where grasses, acorns, chestnuts and other local resources contribute to the origination of the dry-cured ham [5]. These market trends oriented to products originated in alternative production systems, should be cultivated to strengthen the consolidation of the pasture-based pork supply chain and its related niche markets. A market-driven approach could include labelling [6]. In Europe exist legal certifications that allow the identification of certain attributes in products oriented to specialized niche markets. Certifications such as IGP (Indication of Geographic Protection) and DOP (Protected Designation of Origen) indicate that quality traits of the product are linked to the place of production, processing or preparation, and that specific age-old traditional protocols have been followed [7]. In these niche markets, sustainable practices that integrate elements relevant to the environment, animal welfare, food safety, and economic viability are drivers for innovation.

To improve the productivity and animal welfare in pasture pig systems, it is convenient to organize and manage the herd in functional groups according to their productive stage (breeding, gestation, farrowing, growers and finishers) [8,9]. Pasture use is an ideal option for managing gestating sows because older animals can make better use of nutrients from forages, and it provides opportunity for a wide range of favorable behavorial activities. [10]. Additionally, pasture helps alleviate hunger in sows fed a restricted diet at maintenance levels [11]. Pregnant sows grazing *Lolium perenne* and *Trifolium repens* pastures, and managed under supplemental feed restriction strategies presented daily forage intake in the range of 3.4 to 8.2 kg fresh forage + 1.5 kg of supplemental feed, and 6.5 to 8.1 + 3.0 kg of supplemental feed per sow, during spring and summer, respectively [12]. Similarly, lactating sows grazing the same grass and clover mixture but receiving feed supplement *ad libitum* showed lower fresh forage intake (0.2 to 1.6 kg sow^−1^ d^−1^) [13].

Reduction of environmental impact is one of the major goals of sustainable livestock production and imposes underlying pressure to farmers. Indeed, farmers face the challenge of how simultaneously integrate environmental goals with production system practices oriented to achieve animal welfare, farm profitability and consumers’ health. Key elements of a successful grazing system include identifying how many animals to incorporate and defining the grazing period that will minimize the environmental impact on the resources. Concentrating too many animals could damage the vegetative ground cover leaving the soil vulnerable to the influence of rainfall, wind and temperature [14].

Best management practices are designed to support farmers in the search of more sustainable ways to produce food. But this goal is not easily achieved with grazing pigs which, if left uncontrolled, could express potentially environmental damaging behavior such as selective grazing, rooting and the use of preferred dunging areas [15]. Combined with the physical effect of their hooves, swine natural behavior could deteriorate the ground cover, and increase bare soil, soil compaction, erosion, and run-off, thus negatively impacting surface and ground water and decreasing pasture production and productivity [16,17]. Additionally, the grazing of pigs involves a high import of nutrients into the system through the feed. Grazing animals deposit manure and urine in certain sections of the paddocks, therefore if the amount of nutrients deposited surpasses the vegetation capacity to use these nutrients there is a risk of environmental pollution [17]. The amount of nutrients that leave a pasture system through erosion, runoff and leaching can be reduced by maintaining an appropriate ground cover, a factor directly linked with the stocking rate and length of access. Indeed, lower ground cover [18,19,20] and higher soil structural damage [17,21,22] have been related to increased stocking rate.

There are many factors to consider when determining suitable stocking rate for swine on pastures (soil, forage type, weather conditions, animal physiological stage, and management skill) [17]. The effects of stocking rates (ranging from 8 to 92 sows ha^−1^) has been documented [18,19,23,24,25,26,27,28,29,30]. In some European countries, the stocking rates are established based on the projected annual excretion of N (140 kg N ha^−1^) [30]. To our knowledge, the rotational stocking management system as used in this work has not been previously evaluated in terms of ground cover maintenance or soil nutrient upload. Ground cover maintenance and nutrient management are key elements to reduce the environmental impact of outdoor swine production [31]. Maintaining a ground cover greater than 75% and controlling nutrient loading and distribution are considered as best management practices for pasture pig operations [32]. These best management practices improve soil health and water quality, by minimizing runoff, soil erosion and water pollution. This study was conducted to evaluate the effect of three stocking rates (10, 15 or 25 sows-gilts ha^−1^) on ground cover and soil nutrient concentrations of bermudagrass (*Cynodon dactylon* L. Pers) paddocks managed in a weekly rotational stocking system.

## 2. Materials and Methods

### 2.1. Study Area

The research was conducted at the Center for Environmental Farming System (CEFS) Field Research, Education, and Outreach Facility at Cherry Research farm, in Goldsboro (35.38291° N, –78.035846° W), North Carolina (USA). The climate is humid subtropical (Trewartha climate classification), with average temperatures between 10.8 and 22.5 °C and yearly precipitation of 1465 mm. The soils of the experimental site had been classified as Johns sandy loam (Fine-loamy over sandy or sandy-skeletal, siliceous, semiactive, thermic Aquic Hapludults), and Kenansville loamy sand (Loamy, siliceous, subactive, thermic Arenic Hapludults), based on US Soil Taxonomy [33]. The terrain is mostly flat with 0 to 2% slope. The CEFS swine herd is managed in hoop houses using a deep bedding system. The nucleus of the herd is Yorkshire, and has been kept antibiotic-free for almost 40 years, thus requiring that very strict biosecurity measures must be followed. These biosecurity measures restricted the availability of animals during the calendar year, thus constraining the timing of the experiment. The field experiment was conducted over three grazing periods of 8 weeks each during two consecutive years: January to March of year 1; September to November of year 1; and April to June of year 2. A 24-week rest period was implemented between each grazing period.

### 2.2. Animals

The study was conducted in accordance with the animal use and care guidelines of North Carolina State University Institutional Animal Care and Use Committee (IACUC 09-021-A). A total of 60 pure Yorkshire sows and gilts were allotted to one of the three treatments according to pre-experimental individual live weight to balance live weight across stocking rates. Three different groups of animals varying in initial live weight (294.2 ± 8.4, 211.9 ± 6.9 and 186.4 ± 3.0 kg for animals used in grazing period 1, 2 and 3, respectively) and parity were used. Live weight was estimated by weighing the sows when they were turned in and out of the paddocks. The change in live weight during the graze periods was equivalent to 1.2 ± 4.36, 5.58 ± 3.03 and 5.51 ± 2.87 kg for animals included in grazing period 1, 2 and 3, respectively).

Prior to the experimental period, sows-gilts were kept in straw-bedded hoop barns, but were given two weeks of acclimation to a wooded outdoor paddock with an electrical fence. At the beginning of each grazing cycle, animals were arranged in groups of two, three or five corresponding to the treatments. These groups were randomly assigned to the paddocks where they stayed for the 8-week grazing cycle.

**Figure 1:**
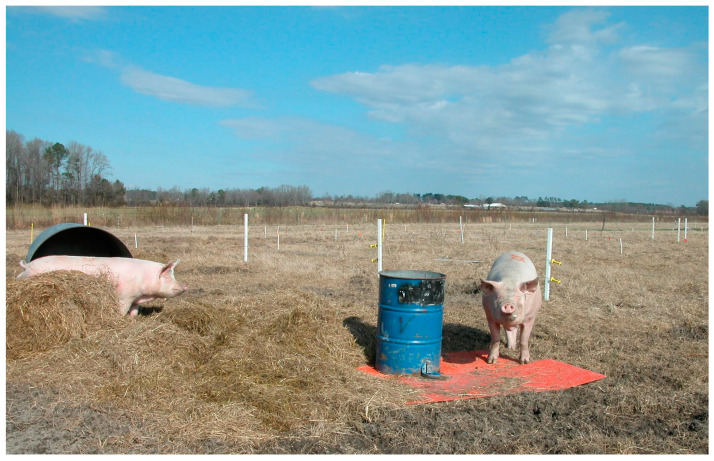
The service areas with the shelter and a water barrel functioning as a drinker were located in the center of the paddocks. A PVC-coated metal slat was placed under the barrel to reduce damage to the soil structure. (January to March, year 1).

**Figure 2:**
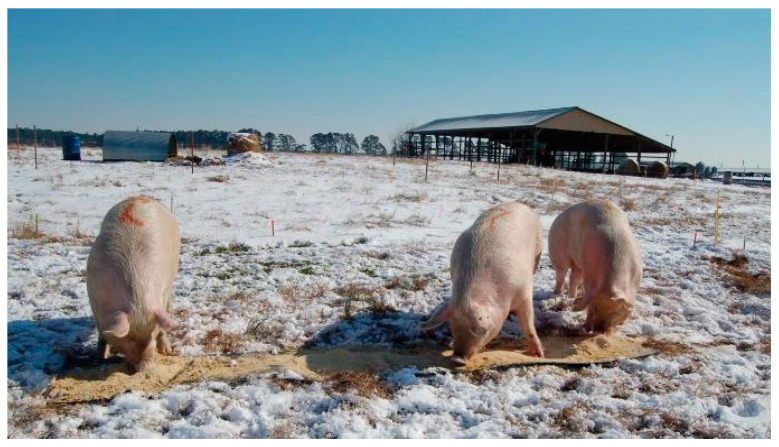
Mats from a repurposed rubber conveyor belt were used daily to offer a farm-made grain mixture. (January to March, year 1). The snow is not frequent in the area and only lasted few days.

During the graze period the animals had free access to shelter and water drinkers located in the center of the paddock (Figure 1). Shelters were built with repurposed metal grain bins open on one end. Sows were offered daily in the morning a home-mixed feed containing corn, soybean, minerals and vitamin mixes. Feed was allocated according to maintenance requirements which averaged 2.0 kg grain mixture head^−1^ d^−1^ plus an additional adjustment of 0.41 kg per every 45 kg of live weight over 180 kg. During the first grazing period when the environmental temperature declined to 12.8 °C, animals received an additional 0.20 kg head^−1^ d^−1^ per 5.6 °C decline in temperature. Feed was analyzed for chemical composition by the North Carolina Department of Agriculture and Consumer Services-Farm Feed Testing Service laboratory. On average, the grain mix contained 161.1 g kg^−1^ crude protein; 43.3 g kg^−1^ crude fat; and 44.5 g kg^−1^ ash (8.7 g kg^−1^ Ca; 5.3 g kg^−1^ P; 2.0 g kg^−1^ S; 1.7 g kg^−1^ Mg; 1.8 g kg^−1^ Na; 8.4 g kg^−1^ K; 163 g kg^−1^ Cu; 281 ppm Fe; 51 ppm Mn; and 139 ppm Zn); net energy was calculated as 2436 kcal kg^−1^ DM. The daily feed was delivered once day^−1^ onto mats made from rubber used conveyor belts providing space enough to allow simultaneous access to feed (each sow had 1 lineal m of the mat available, Figure 2).

### 2.3. Pasture Management

**Figure 3:**
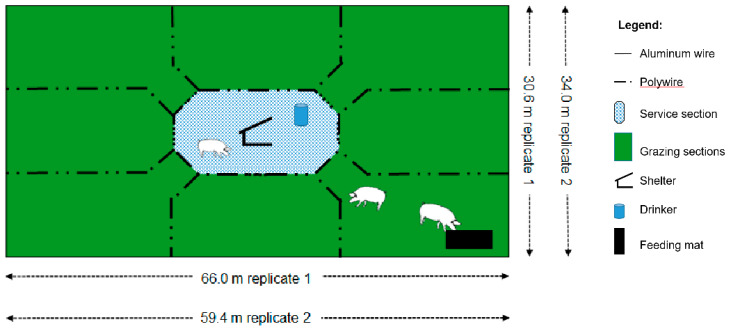
Experimental setup. Paddock dimensions in the field’s replicates were slightly different. (Replicate 1: 30.6 m × 66.0 m and Replicate 2: 34.0 m × 59.4 m). The figure is not drawn to scale.

**Figure 4:**
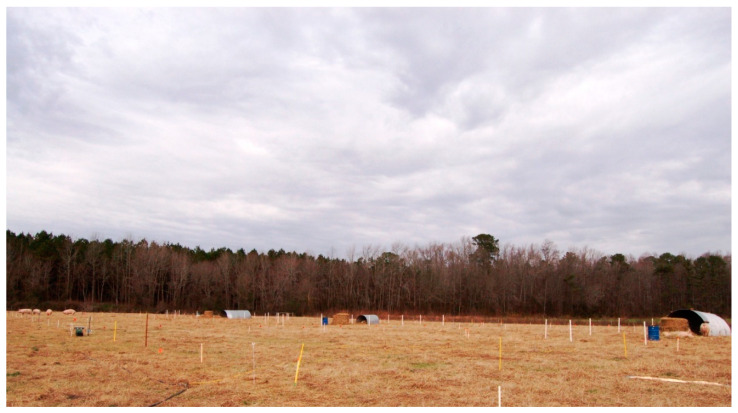
View of one field replicate with three paddocks. The service areas can be identified with the shelters, the barrel-drinkers, and piles of hay. At the far left, four sows in a graze subplot. (January to March, year 1).

A 1.25-ha pasture established with Coastal bermudagrass was divided into two blocks, which were further divided into three rectangular paddocks each. The paddocks differed slightly in dimensions among the two blocks (59.4 m × 34 m vs. 66.0 m × 30.6 m, for block 1 and 2, respectively), and measured an average 2019.6 m^2^. Each paddock was divided using plastic step-posts and electrified poly-wire fencing into nine equal-sized sections (Figure 3; Figure 4).

**Figure 5:**
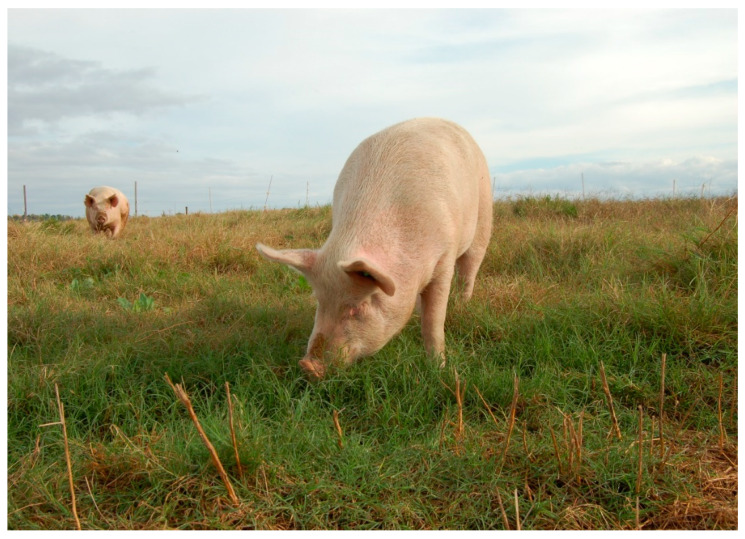
Gilts in a graze section (September to November, year 1).

The central section (representing 11.1% of the paddock, 224.4 m^2^) was considered the service section where shelters and water drinkers were located. The other eight sections were managed as graze sections resulting in an eight subplot rotation (Figure 5). Animals had permanent access to the service sections, and simultaneously to one of the grazing sections on a weekly basis. Each of the graze sections was grazed once (7 d of occupation) per grazing period. In the graze section currently in use, the feed was provided over used conveyor belts acting as feeders. The location of these “feeders” changed daily within the respective section. The water was supplied using metal barrels with attached water-cup drinkers. A PVC-coated metal perforated slat (61 cm × 122 cm, 3/8” openings) was placed under each drinker. The stocking density was equivalent to 10, 15 and 25 sows-gilts ha^−1^ (224.4, 149.6 and 89.8 m^2^ sow-gilt^−1^ week^−1^, respectively; Table 1). The paddocks were managed with the same stocking rate treatments during the three grazing periods. Temporary electric fencing was used to control access to the designated sub-section each week. Each of the 8 sub-sections was grazed for 7 days of the 56-day grazed period. Grazing periods were calendar-fixed with a 24-week rest period among consecutive grazing periods. No fertilizer was applied during the course of the experiment.

### 2.4. Ground Cover Estimation

Ground cover estimation was conducted weekly using a modified step point method [34]. The ground cover was differentiated into three components: live vegetation (including grasses, forbs, leaves and stems), dead-dormant vegetation (litter-plant residues, standing dormant and senesced or dead vegetation) and bare soil. To assess ground cover, each paddock was marked with 24 equally spaced transects using pieces of PVC pipes (70 cm long by 1.27 cm diameter). Every other step along the transect represented a sampling point, in each of which the ground cover was characterized and recorded. On average, 28 sampling points were recorded per transect providing 672 points within each treatment paddock. Ground cover was calculated with the following equation: Ground cover = live vegetation + dead-dormant vegetation [34]. Ground cover was determined weekly, but only data corresponding to the last week (8th week) of each grazing period were included in the statistical analysis.

### 2.5. Soil Samples

Soil samples were collected and assembled before starting the experiment, and following the removal of the animals from the paddocks after the first and third grazing periods. Paddock portions showing evidence of feed waste and manure deposits at the soil surface were excluded in the sampling process. The initial sampling was conducted using a hand probe (2-cm diameter) to a sampling depth of 30 cm. Five soil cores were taken from each of the nine sections of the paddocks, combined (45 cores) and mixed to form one composite sample per paddock (n = 6). One week after the animals were removed at the end of the first grazing period, soil samples were collected using a hand probe to two depths: 0 to 15 and 15 to 30 cm. Each sample was made up of 15 soil cores randomly gathered from each paddock section. Those samples were later combined and mixed per depth to yield a sample corresponding to the service section, and a sample for the graze section (this last one, containing 20 cc subsamples from each of the eight grazing sections). A total of 24 samples were sent to the laboratory. Following the third grazing period (nine days after animal removal) one core soil sample was taken from the center of each of the nine sections of the paddock using a soil coring device (3.2 cm diameter) installed on a truck (Model GSRPS Giddings Machine Company, Windsor, CO). These samples were collected to four sampling depths (0 to 15, 15 to 30, 30 to 60 and 60 to 90 cm) resulting in 216 samples.

Soil samples were air-dried at room temperature, gently crushed, sieved through a 2-mm mesh [35] and sent to the North Carolina Department of Agriculture and Consumer Services Agronomic Division, Soil Testing Section laboratory for standard analysis using Mehlich-3 extraction methodology (Mehlich buffer acidity) for Ca, P, K, Mg, Na, S, Cu, Mn, Zn, and Fe. Samples were also analyzed for ammonium (NH_4_^+^) and nitrate (NO_3_^-^) concentrations (1 M KCl soil extracts analyzed on a Quick Chem 8000 LACHAT) [35] at North Carolina State University Environmental and Agricultural Testing Service laboratory. The amount of nutrients imported to the system through animal feed were estimated.

### 2.6. Experimental Design and Statistical Analysis

A randomized complete block design with a split–split plot arrangement of treatments was used to analyze ground cover data, while soil data were evaluated employing a split plot complete block design with a factorial treatment arrangement. For ground cover, the main plot factor was the grazing period (January to March, year 1, September to November, year 1 and April to June, year 2), stocking rate (10, 15 and 25 sows-gilts ha^−1^) was the subplot factor and section of the paddock (grazing and service sections) represented the sub-subplot factor. Two independent analyses were conducted for the soil samples collected after the first and the last (third) group of sows. For soil nutrients, stocking rate was considered as the main plot factor and soil sampling depth (0 to 15 and 15 to 30 cm; or 0 to 15; 15 to 30; 30 to 60 and 60 to 90 cm, for the sampling conducted after the first or after the third grazing period, respectively) as the subplot factor. Treatments were randomly assigned to the paddocks and each paddock was considered as the experimental unit. All treatments had two field replicates (blocks).

Statistical data analyses were performed with the software SAS version 9.4 (SAS Institute Inc., Cary, NC, USA) using the PROC GLIMMIX (generalized linear mixed models) [36]. In the models for ground cover, grazing period, stocking rate, section of the paddock and their interactions were treated as fixed effects. Blocks and its interactions with grazing period, stocking rate and section of the paddock were considered random effects. In the models for soil nutrients, block and its interactions with stocking rate and sampling depth were included as random effects and stocking rate, sampling depth and their interaction as fixed effects. Differences were considered significant when *p* ≤ 0.05, and *p* ≤ 0.10 were noted as a tendency. Mean separation was conducted using least-squares means including the ADJUST and SIMULATE options.

## 3. Results

**Figure 6:**
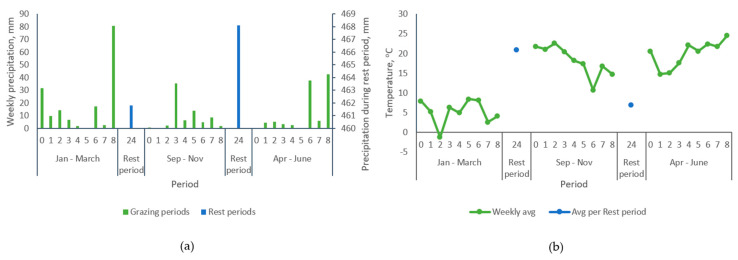
Cumulative precipitation (**a**) and average temperature (**b**) registered during the experimental period. Source: CHRONOS database - State Climate Office Of North Carolina [37] and own estimation.

The cumulative precipitation and average daily temperature recorded during the experiment are presented in Figure 6. The precipitation was 164.2, 73.5 and 101.70 mm for grazing cycles 1, 2 and 3, respectively. Similarly, the average daily temperature for the same periods were 5.16, 18.2 and 19.9 °C. In addition, the amount of precipitation registered during the first and second rest periods were 461.80 mm and 468.10 mm, respectively, and the temperature varied from 21.0 to 7.0 °C during those time intervals. Accordingly, the weather conditions during the experimental period did not have a negative impact on bermudagrass growth and survival. Soil disturbance, vegetation damage and leaching of nutrients can be highly related to soil moisture conditions. Only during week eight of the first graze period was the rainfall sufficient to have potentially impacted those parameters.

### 3.1. Ground Cover

**Figure 7:**
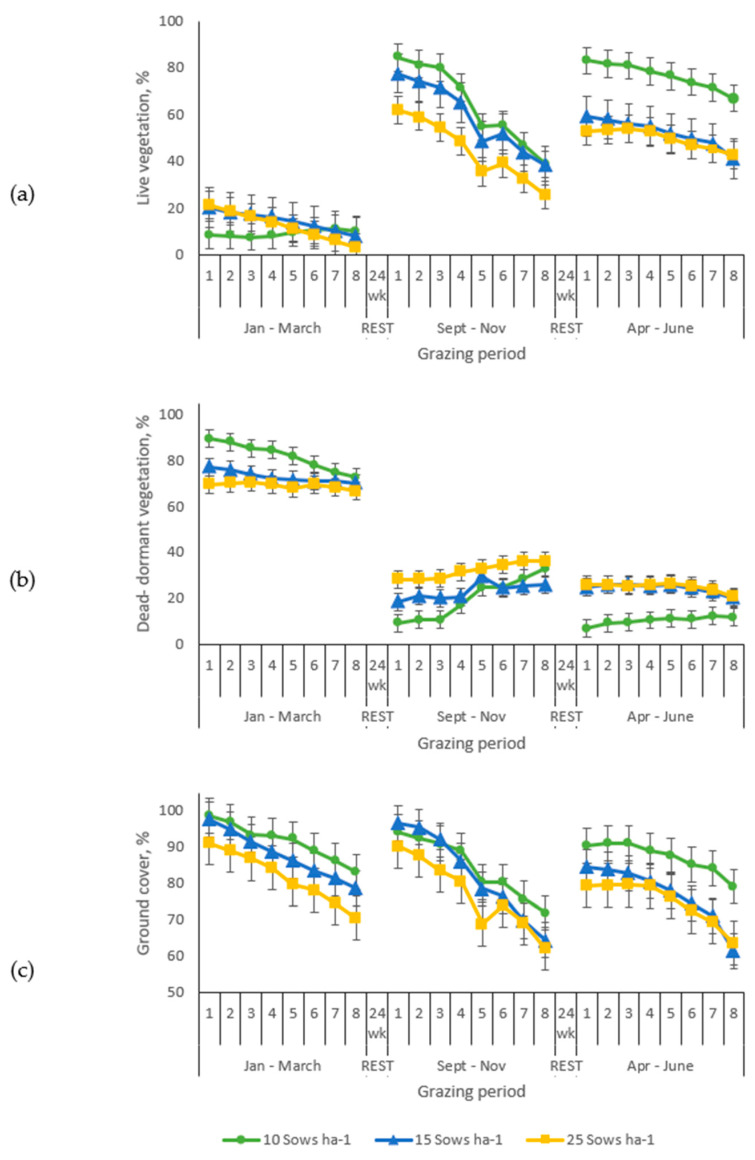
Evolution of the vegetation and the ground cover in the grazing section of bermudagrass paddocks managed with sows-gilts during three grazing periods. (**a**) Live vegetation, (**b**) dead-dormant vegetation, (**c**) ground cover.

This study monitored ground cover changes over time in graze and service areas of the paddocks. Ground cover in the graze sections of the paddocks generally declined weekly throughout each of the grazing cycles, following a similar pattern for all the periods (Figure 7).

During the first two grazing cycles, the ground cover within the graze sections was similar for all stocking rates. However, in the third period, there was less cover in paddocks managed with 15 or 25, compared to those managed with 10 sow ha^−1^. The mean ground cover values during the experimental period were greater than 65%, even for the paddocks managed with the higher stocking rate. The rest period (168 days) between grazing cycles allowed the ground cover to recover in all the paddocks, reaching 93.8 and 85% at the beginning of the second and third period, respectively.

**Figure 8:**
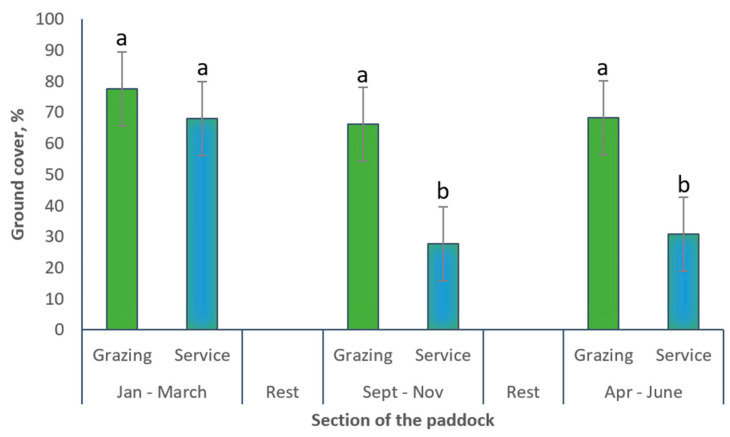
Final ground cover (%) estimated after animal removal in grazing and service sections of bermudagrass paddocks managed with sows-gilts during three grazing periods.

At the end of each grazing period the ground cover (in both graze and service areas) was affected by the grazing cycle (*p* = 0.0011), the stocking rate (*p* = 0.0423), the paddock section (graze or service) (*p* < 0.0001) and the interaction of grazing cycle x paddock section (*p* = 0.0514) (Table 2, Figure 8). Ground cover was 72.6% at the end of the first grazed cycle, but only 48.3% at the end of the 2nd and 3rd graze cycles (47.1 and 49.6%, respectively). Paddocks managed with 10 sows-gilts ha^−1^ had significantly more (35.4 %) ground cover than paddocks with 25 sows-gilts ha^−1^ (Table 2). However, there were no significant differences in ground cover between the stocking rates 10 and 15 sows-gilts ha^−1^ (average 60.7%). The cover was similar also for 15 and 25 sows-gilts ha^−1^ (average 52.1%). The graze section of the paddocks had 68% more ground cover than the service sections (Table 2) except at the end of the first grazing period, when no differences in ground cover were detected among both sections of the paddocks which averaged 72.8% (Table 2; Figure 8). The interactions grazing cycle x stocking rate, stocking rate x paddock’s section, and grazing cycle x stocking rate x paddock’s section were found to be not statistically significant (*p* > 0.05).

### 3.2. Soil Nutrients

Nutrient concentrations in the soil profile following the first 8-week period were not significantly (*p* > 0.05) affected by stocking rate (Table 3). Nevertheless, concentrations of most soil nutrients were highest (*p* < 0.01) in the upper layer (0–15 cm), with the exceptions of P, NH_4_^+^ and Na. However, NH_4_^+^, B and Na tended to decrease with depth (*p* = 0.0843 and *p* = 0.0630, respectively). Additionally, the interaction of stocking rate x soil sampling depth did not influence (*p* > 0.05) either of the soil nutrients levels.

Significant differences in nutrient concentrations following the third graze period were found among stocking rates for NH_4_^+^ (*p* = 0.0082), NO_3_^−^ (*p* = 0.0177), P (*p* = 0.0002), K (*p* = 0.0544), Zn (*p* = 0.0001) and Cu (*p* = 0.0017) (Table 4). The lowest soil nutrient levels were found in paddocks grazed with 10 sows-gilts ha^−1^, with exception of NO_3_^−^, P, Zn and Cu which were not significantly different between 10 and 15 sows-gilts ha^−1^. The soil concentrations of K found in paddocks managed with 25 sows-gilts ha^−1^ were not different for those registered in paddocks managed with the other two stocking rates. Soil nutrient concentrations generally declined with depth (NH_4_^+^, *p* = 0.0002; ***P***, *p* < 0.0001; K, *p* = 0.0013; Ca, *p* < 0.0001; Mg, *p* < 0.0001; S, *p* = 0.0025; Mn, *p* < 0.0001; Zn, *p* < 0.0001; and Cu, *p* < 0.0001), with a general tendency for higher concentrations in the 0 to 15 cm layer. Conversely, NO_3_^−^ concentrations were similar at all sampling depths. Soil concentrations of Na and Fe were not affected by stocking rate nor sampling depth. The initial soil P level in the upper 30 cm was high (1059 kg ha^−1^) and similar (1020 kg ha^−1^) at the end of the experiment. There was not an interaction for stocking rate x sampling depth (*p* > 0.05) following the third graze period.

### 3.3. Estimated Excretion of Nutrients into the System

The estimated amount of N and P imported to the system from the feed over the course of the three grazing cycles are presented in Table 5. The estimated N inputs ranged from 113 kg ha^−1^ (10 sows-gilts ha^−1^) to 280 kg ha^−1^ (25 sows-gilts ha^−1^). Phosphorus input ranged from 23 to 58 kgs ha^−1^ for the 10 to 25 sows-gilts ha^−1^, respectively.

## 4. Discussion

The adoption of the appropriate stocking rate for a pasture system is a critical management decision because it influences animal performance, long-term farm productivity, economic outcomes, wildlife and natural resources. The impact by grazing animals is the result of diverse interrelated comportments including defoliation, treading, trampling, and manure deposition [38]. The implementation of high stocking rates could magnify the undesirable impacts of grazing animals [38]. Previous research has shown the importance of implementing a suitable stocking rate to limit the extent of the damage that grazing pigs could exert to the vegetation and the soil [18,39,40]. The eight-week rotation scheme as evaluated fits in systems where sows-gilts are group-managed in breeding paddocks and moved to gestation paddocks after the second gestation control conducted after the fifth week following mating (38–42d) [41], or in farrowing–lactating paddocks where sows are moved in one week before the farrowing date and removed after weaning [31].

### 4.1. Ground Cover

Maintaining ground cover in outdoor pig systems is critical to controlling water, soil and nutrient movement off site. In these production systems service areas (feeding, watering, housing) receive the greater impact of the pigs. Therefore, it is necessary to manage the paddock in such a way that those service areas can be buffered by grassed areas to minimize runoff and erosion. The morphological and physiological traits of bermudagrass, a warm season perennial species adapted to different types of soils and climates, that shows aggressive growth, good grazing tolerance, and ability to produce biomass of good quality forage when soil nutrients are not limiting [42], make this grass suitable for this kind of systems. Its growth habit and reproductive mechanisms (rhizomes, stolons, and extensive root mass) allowed it to endure the grazing, rooting, trampling and lounging behaviour of the sows, and to recover during the rest periods between successive grazings. The final ground cover reported after the three grazing periods (72.6, 47.1 and 49.6% for the first, second and third grazing periods, respectively) demonstrates the potential of bermudagrass inclusion in pasture-based pig systems. The final ground cover, however, was lower than the 75% recommended to minimize runoff and erosion [32,43]. The first period (January to March) coincided with winter months when bermudagrass is dormant. During that season, most of the ground cover consisted of dead-dormant vegetation (69.1%, Figure 7) which was not nutritionally valuable nor palatable, thus limiting sows foraging behavior and their impact on the vegetation. Besides, pigs tend to decrease their activity during the coldest days of winter as a saving-energy strategy [44]. Additionally, the rooting behavior could have been restricted by soil hardness [45,46,47]. Dormant winter rhizomatous and stoloniferous vegetation (bermudagrass) appears to provide suitable ground cover, even in service areas where animals lounged and drank for eight consecutive weeks. Conversely, the second (September to November) and third (April to June) grazing periods corresponded to fall and spring when the live vegetation accounted on average for 25.8% of the ground cover at the end of each cycle. This fact could have elicited the foraging behavior of the sows attracted by the live vegetation. Softer soil conditions, as a consequence of rainfall, could have made rooting easier [48,49,50]. Additionally, warmer temperatures could have triggered more rooting for thermoregulation purposes, increasing paddock surface damage [51]. Moreover, it has been noticed that pigs tend to impact already affected areas and enlarge existing bare soil spots. What have been called “grazed patches perpetuating”, showing the lack of random area utilization [52]. Conversely, a greater amount of grass cover during summer than during winter (80 vs. 50%) has been reported in paddocks managed with stocking rate of 32 sows ha^−1^ indicating the existence of a seasonal pattern [53], and highlighting the effect of local (soil, forage, management) and climatic conditions.

Some authors [54] suggest expressing stocking rates in terms of kg of live weight ha^−1^ instead of the number of animals ha^−1^ when analyzing the impacts of grazing animals on the grassland ecosystem. In the current study, the heavier animals were used in the first grazing cycle (38% and 58% heavier than animals in the second and third grazing cycle, respectively), during winter months and showed the greater final ground cover (72.6 vs. 48.3%). In spite of each grazing period corresponded to different season, there was no significant interaction between grazing cycle and stocking rate for ground cover, which decreased from 65.0 to 48.0% for the lowest and the highest stocking rates, respectively. Similar results have been reported for different forages species [18,48,55,56].

In the study herein, differences in ground cover were detected among the sections of the paddocks, with graze areas showing 68% more ground cover than the service sections, which represented 11.1% (1/9) of the paddock area. The lower ground cover of the service sections could represent higher risks for runoff and nutrient leaching. The greater impact on the ground cover observed in the service sections is attributed to the permanent access of sows to this area of the paddocks where drinkers and shelters were located. Conversely, the access to one of the eight graze and feeding sections was sequential, and allocated on a weekly basis determined by the rotation. The grazing sections were occupied only one week during each grazing cycle. It has been previously reported that sows show a tendency to concentrate their activities to the areas adjacent to shelters and huts [39,57]. Estimated weighted averages showed an overall ground cover of 67.5% for the paddocks under evaluation across both sections (service and graze areas) and the three grazing cycles. Similarly, weighted average of final ground cover considering both sections of the paddocks showed that only paddock managed with 10 sows-gilts ha^−1^ were able to reach the 75% ground cover conservation goal (75.3, 65.8 and 61.6% final ground cover for 10, 15 and 25 sows-gilts ha^−1^, respectively). To maintain 75% cover on the entire paddock would require an average of 79% ground cover on the 8 graze sections. The rotational system tested in this experiment located the service area in the center of the paddock surrounded by the grazing paddocks, thus minimizing potential runoff that could escape from the service section (Figure 3).

### 4.2. Soil Nutrient

It could be expected that pastures with a greater number of grazing animals would show an increased probability for manure deposition and in consequence greater soil nutrient loads [58]. The sows grazing in the first grazing cycle (January to March) received a greater allocation of feed. They were bigger than the animals used in the two other grazing cycles, but also were supplied additional feed for thermoregulation to compensate for lower environmental temperatures. However, no differences in nutrient content among soil samples from paddocks managed with different stocking rate was noticed in the soil samples collected after removal of the first group of grazing sows. Differences were only observed among samples collected at different depths showing greater accumulation of nutrients in the upper soil layer, except for NH_4_^+^; P and Na, which did not present differences along the soil profile. Conversely, Fe values were 13% higher in the bottom (15 to 30 cm) layer (Table 3). The paddocks responded similarly to management with different stocking rates and did not show a significant impact of stocking rates on soil nutrients concentrations. The differences in amount of feed and consequently in excretion may have been balanced by bermudagrass uptake [18]. The lack of a more marked effect of stocking rates could also be attributed to the rotational stocking system implemented, which allowed the grass a rest period to recover after one week of grazing, and may have led to better nutrients distribution and utilization [59]. Similarly, it may also be possible that the time lapse between manure deposition during the grazing period and the soil sampling date was not long enough to allow the mineralization of organic matter to take place. As the first grazing period was in winter time, the natural decomposition processes could have been delayed [60]. Similarly, an increase in extractable phosphorus in soils managed with lactating sows for six months it has been reported, P levels kept rising even after animal removal [39].

In contrast, the samples collected in the study herein after the third grazing period differed in soil nutrient concentrations according to stocking rates (Table 4). Paddocks managed with 25 sows-gilts ha^−1^, showed greater soil concentrations of NH_4_^+^; NO_3_^-^, P; Zn and Cu (46.7, 128.6, 52.9, 84.3 and 28.6% more, respectively) than paddocks managed with 10 sows-gilts ha^−1^. As these nutrients are major components of pig manure, it may be assumed that the variation of these nutrients detected among the paddocks could be reflecting additional manure deposited in the paddocks that were more intensively managed. Paddocks managed with the higher stocking rates received more nutrients to the system via animal feed and mineralization of the soil organic matter [58]. The presence of greater (35%) ground cover in paddocks managed with the lower stocking rate (10 sows-gilts ha^−1^) could have partially contributed to the reduction in the soil nutrient levels as a consequence of plant uptake [19].

Similarly to the findings of the previous set of samples, the nutrient concentrations varied also according to sampling depth (Table 4) with greater concentrations of NH_4_^+^, P, K, Ca, Mg, S, Mn, Zn and Cu in samples collected from the upper soil strata (0 to 15 cm). The amount of P and K did not vary in the first (0 to 15 cm) and second (15 to 30 cm) layers of soil but showed higher values than in deeper soil strata. However the soil P concentrations in the 30 to 60 and 60 to 90 cm layers (315.7 and 161.6 mg kg^−1^, respectively) are high enough [61] to be considered as evidence of movement through the profile. The relatively low input of P over the course of the three graze cycles resulted in no differences in soil P between the first two layers of soil. While being grazed, the additional organic matter deposited on the soil surface will eventually decay and release its components in the soil profile, circumstance that could increase the concentration of nutrients in the soil upper layer. The differences among nutrients along the soil layers could be indicating what portions of these nutrients may have moved deeper into the soil profile. Irregular dispersion of soil P concentrations in different areas of paddocks managed with pigs have been previously reported, with greater concentrations of P at the deeper soil layers around feeders, huts and wallows [62]. The results obtained herein are in agreement with previous studies where it was indicated an influence of the stocking rates and soil sampling depth on soil nitrate concentrations [20,60,63].

Perennial forages have been included in nutrient management programs to manage soils fertilized with swine manure. In the southeast of the USA, different cultivars of bermudagrass have shown good potential for this purpose. However, differences in the nitrogen use efficiency among cultivars have been found [42]. Previous studies have shown annual uptake values for this species in the range of 314 to 382, and 44 kg ha^−1^ yr^−1^ of N and P, respectively. The species has also shown potential for the extraction of Ca, K, Mg, Cu, Fe, Mn, and Zn [64]. Although excessive or prolonged application of manure could cause nutrients accumulation in the soil, with increased environmental risks.

In this study, a simplified N and P balance was conducted, under the assumption that the inputs to the systems were represented by the feed offered to the animals and entering the system by nutrients deposition via sow excreta. Similarly, little nutrients were expected to leave the system because no major changes were observed in animal weight, and there was no removal of bermudadgrass biomass from the paddocks. Under these conditions, it could be expected a surplus of N close to the values of the imported N. The estimated N inputs ranged from 113 kg ha^−1^ to 280 kg ha^−1^, while P input varied from 23 to 58 kgs ha^−1^ for the 10 and 25 sows-gilts ha^−1^, respectively.

Differences in animal breeds, age, weight, physiological stage, health status, diet, forage species and management, soil type, and environmental conditions make it difficult to make comparisons among systems, and these results are found in scientific literature. Recently, in individual paddocks that included trees and were managed with a stocking rate equivalent to 30 or 37 sows ha^−1^ and seven weeks of occupation, loads of 67 and 78 kg N ha^−1^ respectively, have been reported [31]. However, in research conducted in Texas, there were no differences found in soil nitrate among the tested stocking rates [40].

Outdoor swine production has been associated with an increased risk of nitrate nitrogen leaching [20], especially in nutrient hot-spot areas [18,25,39,65], and deeper than 60 cm in the soil profile. The nitrate values recorded in soil samples collected after the growing season reveal the net balance of N inputs (feed, manure), plant uptake and losses, and represent the amount of nitrate potentially vulnerable to leaching [66]. In this study, NO_3_^-^ levels in the soil profile following the first graze cycle (January to March) (Table 3), were similar for stocking rates, but the concentration in the 0 to 15 cm soil layer was fourfold (7.3 vs. 1.9 mg kg^−1^) higher than in the 15 to 30 cm layer. The NO_3_^-^ concentrations following the first eight weeks of exposure was 93% higher than the initial levels. Bermudagrass was completely dormant during this grazing cycle, therefore nutrient leaching was likely [20,66].

On the contrary, following the third grazing cycle, the paddocks managed with 25 sows-gilts ha^−1^ contained about 113% more NO_3_^-^ than those managed with 10 and 15 sows-gilts ha^−1^, with no effect of soil depth. The NO_3_^-^ concentrations for the 0 to 30 cm soil profile was about 46% of the initial level before sows entered the paddocks. High levels of soil nitrate have been associated with increased N losses [66,67], but the levels of NO_3_^-^ herein reported are unlikely to be of concern [67]. The overall relatively low concentrations of NO_3_^-^ following the third grazing cycle as compared to the first grazing period could be attributed to differences in the temperature among grazing cycles that increase ammonia volatization, NO_3_^-^ leaching, and forage uptake. In soils similar to those of the experimental area, the bermudagrass has an uptake potential of 22 kg N Mg^−1^ DM and 2.7 kg P Mg^−1^ DM [68]. Additionally, the production potential for best management practices on the soils at this site is approximately 11Mg DM ha^−1^ [68]; however, no crop removal nor animal products were removed from the site, therefore N output from the system as bemudagrass biomass was negligible.

The addition of organic-N or NH_4_^+^-N to soils during the nongrowing season presents the risk of leaching into the groundwater [20,66]. In this study, the stocking rate did not affect soil NH_4_^+^ after the first grazing cycle. However, following the third graze cycle NH_4_^+^ was 40.5% higher in paddocks managed with 15 and 25 sows-gilts ha^−1^ than in paddocks managed with 10 sows-gilts ha^−1^, similarly the NH_4_^+^ was 56.5% higher in the 0 to 15 cm soil layer than the layers below. The NH_4_^+^ concentration in the 0 to 30 cm layer was about 61.8% higher than initial levels. The levels observed after the third graze cycle could indicate a threat of potential loss of nitrogen via nitrification [53] or ammonia volatilization [65]. The amount of such losses would be related to climatic factors, vegetation, and other management practices [53]. There appeared to be no differences in soil nutrients between grazed and service areas with the exception of NO_3_^-^ that was 148% higher and K that was 29% higher in the service area, indicating that animals may have been depositing more urine there than in the grazed areas.

Reductions of stocking rate and homogenization of the distribution of the manure are best management practices toward reducing the N surplus in sow pasture-based systems [60,63]. Some studies showed a decrease in soil nutrient levels with the increase of distance to the feeding stations [69,70]. Similarly, the service area of paddocks rotationally stocked during 12 years with 3 sows ha^−1^, exhibited greater bulk density and resistance to penetration, and higher soil concentrations of P, K, and Zn than the areas dedicated to grazing [71,72]. However, contradictory results of the benefits of weekly rotations of shelters, feeders and drinkers on the dispersion of inorganic N in soils has been reported [55]. In the study herein, the feeding mats were located in the currently grazed section of the paddocks, with daily repositioning at feeding time, and shifted to the next grazed section with the animals on a weekly basis, while the shelter and water were permanently located in the central section. The sows were maintained in small areas and recurrent movement of feeders and shelters was performed to create a better distribution of the nutrients in the swine paddocks [39]. It is also important to provide a rest period to the pasture in order to get a regrowth of grass using the nutrients deposited to the soil. Therefore, paddock design and the location of shelters, and feeding and watering points should be taken into consideration for a successful distribution of manure on pasture [31].

A critical issue with the addition of swine manure to soils is the potential risk of increasing the concentration of heavy metals originated from pig feedstuffs and additives [24,69,73]. In the study herein, an increase in the concentrations of zinc and copper in the soil were observed when intensifying the stocking rate from 10 to 25 sows-gilts ha^−1^; nonetheless, the values attained remained under the levels for environmental concerns [74].

An additional benefit of the system implemented in this study is the use of a perennial species of forage to minimize nitrate leaching [42,75]. The larger root biomass of perennial forages and their nutrient uptake reduce leachate under the root zone in comparison with annual crops [76].

## 5. Conclusions

Sustainable pastured pork production systems depend on the implementation of the best management practices, which include (1) applying adequate stocking rates, (2) implementing rotation strategies that match seasonal pasture growth rates, (3) shorter paddock occupation periods, and (4) modifying animal behaviour through the thoughtful placement and movement of feeders in relation to housing and drinking areas.

The rotational system implemented in this study provides insight into management strategies that can be used to intensify the grazing of pasture-based sows and reduce environmental impacts. The combination of perennial forage, higher stocking rates, and short occupation periods lead to maintenance of ground cover and better distribution of nutrients, which could reduce erosion and loss of nutrients. Future research should be geared towards assessing (1) the long-term impact of the proposed stocking rates for sow-gilts or growing pigs and (2) the role of multispecies pastures and feeding strategies in terms of environmental impact.

## Figures and Tables

**Table 1:** Stocking rates for sows-gilts grazing bermudagrass for three grazing cycles of eight weeks in an 18-month period.

Grazing Cycle		Stocking Rate, Sows-Gilts ha^−1^		
		**10**	**15**	**25**
	m^2^ head^−1^	1000	667	400
1st, Jan to March	Kg Live weight ha^−1^	2942	4413	7355
2nd, Sept to Nov	Kg Live weight ha^−1^	2119	3179	5298
3rd, Apr to June	Kg Live weight ha^−1^	1864	2796	4660

**Table 2:** Final vegetative ground cover (%, means and standard error) estimated after animal removal in bermudagrass paddocks managed with sows-gilts during three grazing periods.

Factors	Vegetation (%)		
	Living	Dead-Dormant	Ground Cover
Grazing period			
*p*	<0.0001	<0.0001	0.0011
Jan to March, year 1	3.7 b	68.9 a	72.6 a
Sept to Nov, year 1	25.3 a	21.8 b	47.1 b
Apr to Jun, year 2	26.4 a	23.2 b	49.6 b
SE	±3.7	±8.3	±11.1
Stocking rate			
*p*	0.0948	0.4417	0.0427
10 sows ha^−1^	23.2	41.8	65.0 a
15 sows ha^−1^	18.1	38.2	56.3 a,b
25 sows ha^−1^	14.1	33.9	48.0 b
SE	±3.7	±8.3	±11.1
Paddock section			
*p*	<0.0001	0.4318	<0.0001
Grazing	30.8 a	40.0	70.7 a
Service	6.1 b	36.0	42.1 b
SE	±3.3	±7.9	±10.8

*p*: Probability, SE: standard error. a,b,c: means in a column followed by a common letter are not significantly different by the *t*-test at the 5% level of significance. Stocking rates expressed as kilograms of live weight ha^−1^ are equivalents to 2942, 4413 and 7355; 2119, 3179 and 5298; and 1864, 2796 and 4660 kg LW ha^−1^, for grazing periods 1, 2 and 3, respectively.

**Table 3:** Soil nutrients (mg kg^−1^) in bermudagrass paddocks following the first eight weeks of grazing with sows-gilts (January to March).

Factors	NH_4_^+^	NO_3_^−^	P	K	Ca	Mg	S	Mn	Zn	Cu	Na	Fe
	**(mg kg^−1^)**											
Initial values	8.5	2.4	470.6	193.7	863.8	182.2	17.8	17.1	9.6	1.0	16.8	1081.2
SE	± 1.1	± 0.5	± 76.6	± 24.9	± 75.4	± 18.9	± 1.1	± 4.6	± 3.2	± 0.2	± 0.8	± 108.9
Stocking rate												
*p*	0.5845	0.4513	0.6888	0.3215	0.5459	0.3984	0.3218	0.4859	0.3426	0.2753	0.4162	0.2570
10 Sows ha^−1^	8.5	3.2	419.4	176.3	779.1	162.0	18.3	15.2	6.5	0.8	20.3	1099.0
15 Sows ha^−1^	12.7	5.3	488.3	195.9	888.5	181.8	19.6	18.2	11.6	1.2	25.0	1027.6
25 Sows ha^−1^	10.9	5.3	492.4	213.0	851.6	187.5	21.4	18.0	8.2	1.0	25.4	1335.0
SE	± 2.7	± 1.2	± 171.4	± 25.5	± 64.5	± 11.9	± 1.2	± 10.4	± 4.9	± 0.24	± 2.5	± 10.8
Soil depth												
*p*	0.0843	0.0110	0.6632	<0.0001	<0.0001	<0.0001	0.0050	0.0037	0.0012	0.0025	0.0630	0.0187
0–15 cm	12.8	7.3 a	473.1	238.3 a	976.3 a	212.9 a	21.7 a	19.8 a	11.2 a	1.1 a	25.7	1083.8 b
15–30 cm	8.5	1.9 b	460.4	151.8 b	703.3 b	141.3 b	17.8 b	14.5 b	6.4 b	0.9 b	21.4	1223.9 a
SE	± 2.1	± 1.2	± 164.7	± 24.4	± 40.6	± 8.3	± 0.9	± 10.4	± 4.7	± 0.2	± 1.9	± 67.9
Stocking rate x Soil depth												
*p*	0.7755	0.5102	0.7760	0.6309	0.5528	0.7962	0.6379	0.7953	0.5683	0.4429	0.5663	0.7556

NH_4_^+^: ammonium, NO_3_^−^: nitrate, P: Phosphorus, K: potassium, Ca: calcium, Mg: magnesium, S: Sulphur, Mn: manganese, Zn: zinc, Cu: copper, Na: sodium, Fe: Iron. SR: stocking rate, Soil depth: sampling depth. *p*: probability, SE: standard error. Initial values: Composite samples collected in each paddock before grazing with sows, N = 6. a,b,c: means in a column followed by a common letter are not significantly different by the *t*-test at the 5% level of significance. Stocking rates expressed as kilograms of live weight ha^−1^ are equivalents to 2942, 4413 and 7355 kg LW ha^−1.^

**Table 4:** Soil nutrients (mg kg^−1^, means and standard error) in bermudagrass paddocks following three eight-week periods of grazing with sows-gilts (January to March, year 1; September to November, year 1; April to June, year 2).

Factors	NH_4_^+^	NO_3_^−^	P	K	Ca	Mg	S	Mn	Zn	Cu	Na	Fe
	**(mg kg^−1^)**											
Initial values	8.5	2.4	470.6	193.7	863.8	182.2	17.8	17.1	9.6	1.0	16.8	1081.2
SE	± 1.1	± 0.5	± 76.6	± 24.9	± 75.4	± 18.9	± 1.1	± 4.6	± 3.2	± 0.2	± 0.8	± 108.9
Stocking Rate												
*p*	0.0082	0.0177	0.0002	0.0544	0.1156	0.6400	0.2184	0.6795	0.0001	0.0017	0.649	0.1167
10 Sows ha^−1^	9.0 b	0.7 b	280.6 b	135.9 b	633.0	144.8	14.9	12.6	5.1 b	0.7 b	13.7	1020.3
15 Sows ha^−1^	12.1 a	0.8 b	346.1 b	184.0 a	605.6	138.5	18.5	13.6	5.5 b	0.8 b	14.8	1185.2
25 Sows ha^−1^	13.2 a	1.6 a	428.9 a	162.9 a,b	677.6	147.7	16.7	13.3	9.4 a	0.9 a	14.2	1163.0
SE	± 1.3	± 0.4	± 172.9	± 23.3	± 51.5	± 29.7	± 2.5	± 9.6	± 3.4	± 0.2	± 1.1	± 58.2
Soil depth												
*p*	0.0002	0.9014	<0.0001	0.0013	<0.0001	<0.0001	0.0025	<0.0001	<0.0001	<0.0001	0.8271	0.2910
0–15 cm	15.7 a	1.2	443.5 a	204.5 a	939.9 a	194.9 a	16.3 a	18.1 a	12.8 a	1.0 a	13.8	1012.4
15–30 cm	11.8 b	1.0	486.6 a	176.5 a,b	688.0 b	130.1 b	14.2 b	14.5 b	7.4 b	0.8 b	14.0	1129.3
30–60 cm	9.3 b	1.1	315.7 b	150.9 b,c	535.1 c	131.4 b	13.9 b	11.8 b	4.8 b	0.8 b	14.1	1186.5
60–90 cm	9.0 b	1.0	161.6 c	111.8 c	391.9 d	118.2 b	22.2 b	8.3 c	1.8 c	0.6 c	15.0	1163.3
SE	± 1.4	± 0.4	± 173.4	± 24.6	± 53.4	± 30.0	± 2.6	± 9.6	± 3.5	± 0.2	± 1.2	± 67.2
Stocking rate x Soil depth												
*p*	0.999	0.9911	0.9267	0.9810	0.8897	0.7618	0.8911	0.9792	0.8455	0.8934	0.9976	0.9992

NH_4_^+^: ammonium, NO_3_^-^: nitrate, P: Phosphorus, K: potassium, Ca: calcium, Mg: magnesium, S: Sulphur, Mn: manganese, Zn: zinc, Cu: copper, Na: sodium, Fe: Iron. SR: stocking rate, Soil depth: sampling depth. *p*: probability, SE: standard error. Initial values: Composite samples collected in each paddock before grazing with sows, N = 6. a,b,c: means in a column followed by a common letter are not significantly different by the *t*-test at the 5% level of significance. Stocking rates expressed as kilograms of live weight ha^−1^ are equivalents to 2942, 4413 and 7355; 2119, 3179 and 5298; and 1864, 2796 and 4660 kg LW ha^−1^, for grazing periods 1, 2 and 3, respectively.

**Table 5:** Estimated Total N and Total P (kg ha^−1^) imported to the system via feed offer.

Stocking Rate	Grazing Cycle			
	1st	2nd	3rd	Total
sows-gilts ha^−1^	Nitrogen, kg ha^−1^			
10	50	33	30	113
15	75	49	45	169
25	124	82	74	280
sows-gilts ha^−1^	Phosphorus, kg ha^−1^			
10	10	7	6	23
15	15	10	9	34
25	26	17	15	58
